# The Synergistic Combination of Curcumin and Polydatin Improves Temozolomide Efficacy on Glioblastoma Cells

**DOI:** 10.3390/ijms251910572

**Published:** 2024-09-30

**Authors:** Annalucia Serafino, Ewa Krystyna Krasnowska, Sabrina Romanò, Alex De Gregorio, Marisa Colone, Maria Luisa Dupuis, Massimo Bonucci, Giampietro Ravagnan, Annarita Stringaro, Maria Pia Fuggetta

**Affiliations:** 1 Institute of Translational Pharmacology, National Research Council of Italy (CNR), 00133 Rome, Italy; ewa.krasnowska@ift.cnr.it (E.K.K.); sabrina.romano@ift.cnr.it (S.R.); alexdegregorio24@gmail.com (A.D.G.); gprav@unive.it (G.R.); mariapia.fuggetta@ift.cnr.it (M.P.F.); 2 National Center for Drug Research and Evaluation, Italian National Institute of Health (ISS), 00161 Rome, Italy; marisa.colone@iss.it (M.C.); marialuisa.dupuis@iss.it (M.L.D.); annarita.stringaro@iss.it (A.S.); 3 Association for Research on Integrative Oncology Therapies (ARTOI) Foundation, 00165 Rome, Italy; max.bonucci@gmail.com

**Keywords:** curcumin, polydatin, temozolomide, chemoresistance, glioblastoma

## Abstract

Glioblastoma (GBL) is one of the more malignant primary brain tumors; it is currently treated by a multimodality strategy including surgery, and radio- and chemotherapy, mainly consisting of temozolomide (TMZ)-based chemotherapy. Tumor relapse often occurs due to the establishment of TMZ resistance, with a patient median survival time of <2 years. The identification of natural molecules with strong anti-tumor activity led to the combination of these compounds with conventional chemotherapeutic agents, developing protocols for integrated anticancer therapies. Curcumin (CUR), resveratrol (RES), and its glucoside polydatin (PLD) are widely employed in the pharmaceutical and nutraceutical fields, and several studies have demonstrated that the combination of these natural products was more cytotoxic than the individual compounds alone against different cancers. Some of us recently demonstrated the synergistic efficacy of the sublingual administration of a new nutraceutical formulation of CUR+PLD in reducing tumor size and improving GBL patient survival. To provide some experimental evidence to reinforce these clinical results, we investigated if pretreatment with a combination of CUR+PLD can improve TMZ cytotoxicity on GBL cells by analyzing the effects on cell cycle, viability, morphology, expression of proteins related to cell proliferation, differentiation, apoptosis or autophagy, and the actin network. Cell viability was assessed using the MTT assay or a CytoSmart cell counter. CalcuSyn software was used to study the CUR+PLD synergism. The morphology was evaluated by optical and scanning electron microscopy, and protein expression was analyzed by Western blot. Flow cytometry was used for the cell cycle, autophagic flux, and apoptosis analyses. The results provide evidence that CUR and PLD, acting in synergy with each other, strongly improve the efficacy of alkylating anti-tumor agents such as TMZ on drug-resistant GBL cells through their ability to affect survival, differentiation, and tumor invasiveness.

## 1. Introduction

Glioblastoma (GBL) is a highly proliferative and poorly differentiated primary brain tumor; it is currently treated by surgery and/or radiotherapy and systemic therapy, mainly consisting of temozolomide (TMZ)-based chemotherapy. TMZ is an oral alkylating agent that exerts its cytotoxic effect mainly through the methylation of guanine residues in DNA at the O6 position. This adduct can be removed by the DNA repair protein O6-methylguanine-DNA-methyltransferase (MGMT), which is heterogeneously expressed in glioblastoma and whose gene transcription is epigenetically silenced by the methylation of its promoter. Thus, hypermethylation of the MGMT gene promoter can confer a greater sensitivity of the tumor cell to pharmacological treatment with TMZ, and the MGMT promoter methylation status is currently used as a predictor for the benefit of TMZ therapy [1,2].

Despite recent advances in the above multimodal protocols, the overall prognosis for GBL patients remains poor due to the establishment of TMZ resistance and tumor recurrence through infiltrative and invasive residual cancer cells, with a median survival of <2 years [3]. Thus, additional and innovative therapeutic strategies are needed to improve the outcomes of patients with GBL.

In recent decades, the identification of natural compounds that exert strong anti-tumor activity by acting as potent modulators of cancer-related pathways [4] has led to the design of therapeutic protocols that combine these compounds with conventional chemotherapeutic agents. In particular, since the available therapeutic protocols for GBL treatment are not very effective, over 50% of patients use complementary and alternative approaches, among which, herbal therapies are the most commonly used but, until now, with little success.

Curcumin [(1E,6E)-1,7-bis(4-hydroxy-3-methoxyphenyl)-1,6-heptadiene-3,5-dione] is the main natural polyphenol extracted from the rhizome of *Curcuma longa* L., a flowering plant of the ginger family (Zingiberaceae) [5], which is mainly grown in Asia and Central America. *Curcuma longa* L., also known by the common name of turmeric, is widely used in traditional medicines, such as in traditional Chinese and Ayurvedic medicine, for different kinds of diseases due to its antioxidant [6], anti-inflammatory [7], neuroprotective [8], and antimicrobial properties [9,10]. Furthermore, its anticancer potential has been widely described and is still the topic of numerous studies [11,12,13].

Polydatin (3,5,4-trihydroxystilbene 3-O-beta-D-glucopyranosid, PLD), a glycoside of resveratrol (RES), is a natural compound belonging to the stilbene class in the polyphenol family. It is extracted from the roots and rhizome of *Polygonum cuspidatum*; it was originally from Asia but is currently widespread in America and Europe. It is considered the “twin” molecule of RES, with which, it shares properties but has some additional advantages. Specifically, PLD is more efficiently absorbed by the oral route and is more resistant to enzymatic oxidation than RES. In addition, RES penetrates cells passively, whereas PLD enters cells through an active mechanism based on glucose carriers. These properties make PLD more bioavailable than RES [14,15]. PLD is known to have numerous beneficial effects as an antioxidant [16,17], anti-inflammatory [18,19], and immune-modulating agent [20], and as an anti-tumor natural compound for different kinds of cancer cells, including glioblastoma cells [21,22,23,24,25].

CUR and PLD (or its derivative RES) are widely used alone or in combination in the pharmaceutical field, as drugs, and in the nutraceutical field, as food supplements. Several studies demonstrated that the combination of low doses of CUR and RES had a more potent cytotoxic efficacy than the individual compounds alone on different types of cancer [26,27]; they synergistically decreased cancer cell migration [28], shaped immune responses while reducing cancer cell survival [29], and suppressed chemoresistance [30]. 

In a recent clinical study performed for patent applications (WO-2020026185-A1 and PCT/IB2019/056565) [31], we obtained data supporting the synergistic efficacy of the sublingual administration of a combination of CUR (as a new water-dispersible formulation) plus PLD in the treatment of grade 3 and 4 GBL as a protocol for an integrated anticancer therapy. Specifically, the new nutraceutical formulation of CUR+PLD improved patient survival and significantly reduced the size of the tumor masses. 

These preliminary but stimulating results prompted us to obtain some experimental evidence reinforcing the efficacy of CUR+PLD in GBL patients. To this end, in this study, we assessed if pretreatment with a combination of CUR and PLD can synergistically improve TMZ's anti-tumor efficacy in two human glioblastoma cell lines that exhibit different degrees of responsiveness to this chemotherapeutic drug. The effects on cell viability and morphology, and on proteins related to cell proliferation, differentiation, apoptosis or autophagy, and the actin network were analyzed.

## 2. Results

### 2.1. Establishment of the Dose- and Time-Dependent Responses to TMZ and of the Synergic Concentrations of CUR and PLD for U87 and LN18 Glioblastoma Cells

**Figure 1:**
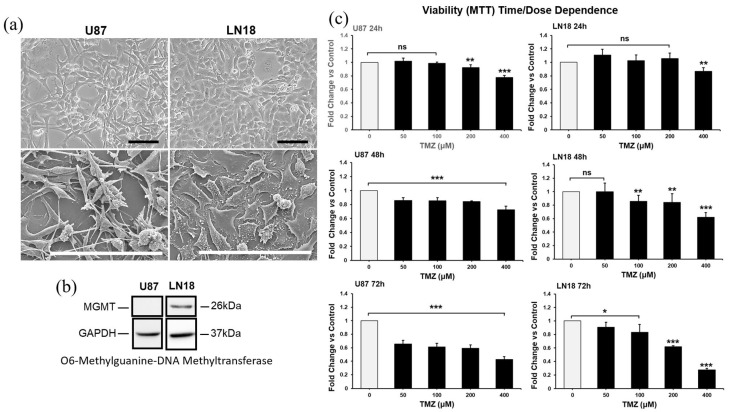
Features of cell lines used (**a**,**b**) and dose- and time-dependent responses to TMZ of U87 and LN18 cells (**c**). (**a**) Phase contrast microscopy (**top** panels) and scanning electron microscopy (**bottom** panels) images illustrating the morphological features of U87 and LN18 glioblastoma cell lines. The scale bars represent 100 μm. (**b**) Baseline expression, measured by WB, of the DNA repair protein O6-methylguanine-DNA-methyltransferase (MGMT) in U87 and LN18 cells. GAPDH was used as the loading control. (**c**) Time- and dose-dependent response experiments performed using the MTT assay to define the optimal condition for TMZ treatment of U87 and LN18 cells. Results are the mean ± SD from three independent experiments (*n* = 3). Significance vs. untreated control (two-tailed Student’s *t*-test): * *p* < 0.05, ** *p* < 0.01, *** *p* < 0.001; ns: not significant.

Given the heterogeneous expression of the DNA repair protein MGMT in GBM, verifying if the combination of CUR+PLD could be of benefit independent from the methylation levels of the MGMT gene promoter and, consequently, from the sensitivity to TMZ, we used, as cellular models of glioblastoma, the cell lines U87 and LN18, which are negative and positive for MGMT expression, respectively (Figure 1b). The phase contrast microscopy (Figure 1a, top panels) and SEM analyses (Figure 1a, bottom panels) showed that the two cell lines exhibited different morphological features, with the U87 cells being mainly neurite-like while the LN18 cells had an epithelial morphology. In the preliminary time- and dose-response experiments, TMZ was used at concentrations ranging from 50 to 400 µM, and the dose of 200 µM for 72 h was selected as the optimal condition for subsequent experiments since this condition reduced cell viability to close to 50% of that of the untreated control in both cell lines (Figure 1c).

To identify the concentrations of CUR and PLD that produce a synergic effect on cell viability, the U87 and LN18 cell lines were treated with CUR and PLD (individually or combined) at concentrations ranging from 0.18 to 10 µg/mL for CUR and 17.5 to 300 µg/mL for PLD, and cell viability was tested after 24 h using the MTT assay. The combination index (CI) for all the concentrations and ratios used was defined by the CalcuSyn software v2.1; the analyzer automatically quantified the synergism or antagonism between the drugs. Supplementary Figure S1 reports some representative results obtained by CalcuSyn in both cell lines tested. The concentrations of 10 µg/mL for CUR and 100 µg/mL for PLD (ratio of 1:10) were selected as the synergic combination to be used in the subsequent experiments since it showed synergism in both cell lines (CI < 0.9); more specifically, it showed a strong synergism in U87 cells (CI = 0.101) and a moderate synergism in LN18 (CI = 0.772).

**Figure 2:**
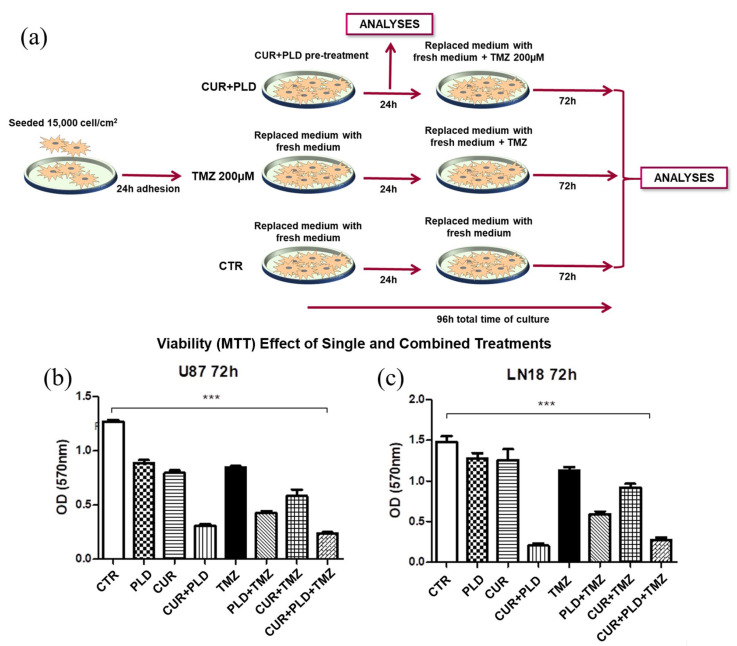
Experimental scheme (**a**) and MTT cell viability assay results (**b**,**c**) from the preliminary assessment of the efficacy of the selected synergic combination of CUR+PLD. (**a**) Scheme of the protocol used for evaluating the effects of treatments on morphology, viability, and protein expression in both cell lines, as detailed in the Methods section. (**b**,**c**) Viability assay (MTT assay) results for U87 and LN18 cells subjected to TMZ treatments in the presence or absence of CUR and PLD as single or combined pretreatment. Values are the mean ± SD from three independent experiments (*n* = 3). Significance vs untreated control (two-tailed Student’s *t*-test): *** *p* < 0.001.

The effectiveness of the selected combination of CUR+PLD in improving the effect of TMZ on cell viability was preliminarily demonstrated using the MTT test (Figure 2b,c), performed following the experimental protocol schematized in Figure 2a, which showed that the pretreatment with CUR+PLD decreased the number of viable cells by more than 50% compared to the treatment with TMZ alone. The synergistic effect was also confirmed by the higher efficacy of the pretreatment with the combination of CUR+PLD vs. those with CUR or PLD alone. Interestingly, for both cell lines, in the samples subjected to a 24 h treatment with CUR+PLD and analyzed after an additional 72 h of culture in fresh medium (72 h washout), a reduced number of viable cells, comparable to that recorded in samples post-treated with TMZ, was recovered (Figure 2b,c).

### 2.2. Effects of the Synergic Combination of CUR+PLD on U87 and LN18 Glioblastoma Cells before TMZ Treatment

**Figure 3:**
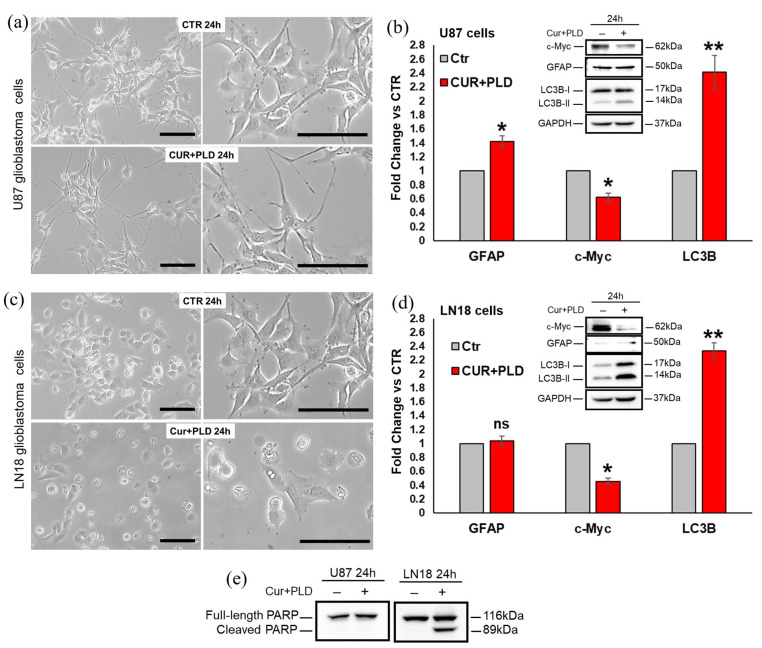
Effects of the synergic combination of CUR+PLD before TMZ treatment on U87 and LN18 cells. (**a**,**c**) Phase contrast microscopy images showing the morphological changes induced by 24 h CUR+PLD treatment on U87 (**a**) and LN18 (**c**) cells. The scale bars represent 100 μm. (**b**,**d**,**e**) WB and analysis of modifications induced by 24 h CUR+PLD treatment on expression levels of the proliferation marker c-Myc, the astroglial differentiation marker GFAP, the autophagosomal marker LC3B and the marker of caspase-dependent apoptosis PARP. Histograms in (**b**,**d**) report the densitometric analysis results for GFAP, c-Myc, and LC3B expression; values were normalized to GAPDH. Results are the mean ± SD from three independent experiments (*n* = 3). Significance vs. control (two-tailed Student’s *t*-test): * *p* < 0.05, ** *p* < 0.01; ns: not significant.

To assess which modifications were induced by the CUR+PLD combination before the TMZ treatment, both cell lines were analyzed, after 24 h of CUR+PLD treatment, in terms of cell morphology and the expression levels of proteins related to cell proliferation (c-Myc), astroglial differentiation (GFAP), caspase-dependent apoptosis (cleaved PARP), and autophagy (LC3B). Compared with the untreated control, CUR+PLD-treated U87 cells exhibited a more marked astrocyte-like morphology, with more extensive and elongated branching (Figure 3a). 

Concomitantly, the WB analysis showed a significant decrease in expression of the proliferation marker c-Myc, a weak but significant increase in the expression of the astroglial differentiation marker GFAP, as well as an increase in the levels of the autophagosomal marker LC3B-II (Figure 3b), whereas the cleaved form of PARP was not expressed, suggesting that 24 h of CUR+PLD did not induce caspase-dependent apoptosis in U87 cells (Figure 3e). Conversely, LN18 cells subjected to 24 h treatment with CUR+PLD did not exhibit an astrocyte-like morphology but showed, in contrast to the untreated control, numerous cells displaying morphological features indicative of apoptosis/autophagy (Figure 3c). The WB analysis (Figure 3d,e) showed that GFAP expression was not modified compared with LN18 control cells (that were almost negative for this astroglial differentiation marker), but the levels of the autophagosomal marker LC3B-II and the cleaved form of PARP were significantly increased, and, similar to what was recorded for U87 cells, the expression of the proliferation marker c-Myc was significantly decreased compared with the untreated control.

### 2.3. Pretreatment with CUR+PLD Significantly Improved the Effect of TMZ Treatment on the Number of Live U87 and LN18 Glioblastoma Cells

**Figure 4:**
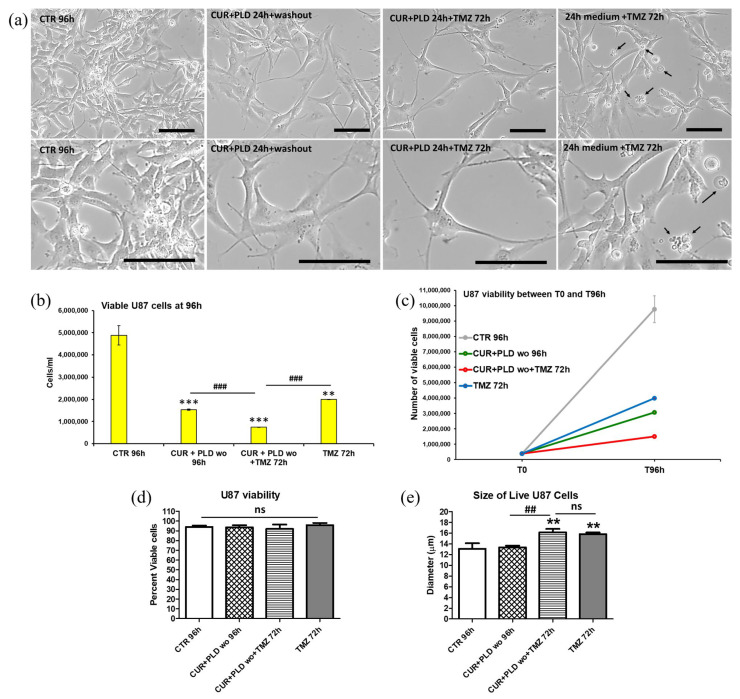
Effects of pretreatment with the synergic combination of CUR+PLD on TMZ-induced effects on U87 cells. (**a**) Phase contrast microscopy images showing the morphological modifications induced in untreated controls and in samples pretreated for 24 h with CUR+PLD with or without TMZ post-treatment. Arrows point to rare cells displaying morphological features suggestive of apoptosis/autophagy. The scale bars represent 100 μm. (**b**–**e**) Analysis results from the automated CytoSmart cell counter: the number of live cells (**b**), cell growth between T0 and T 96 h (**c**), percent viable cells (**d**), and cell size (**e**). Results are the mean ± SD from three independent experiments (*n* = 3). Significance (one-way ANOVA + Tukey multiple comparison test): ** *p* < 0.01, *** *p* < 0.001 vs. untreated control (CTR); ## *p* < 0.01, ### *p* < 0.001 vs. TMZ alone or pretreated with CUR+PLD; ns: not significant.

**Figure 5:**
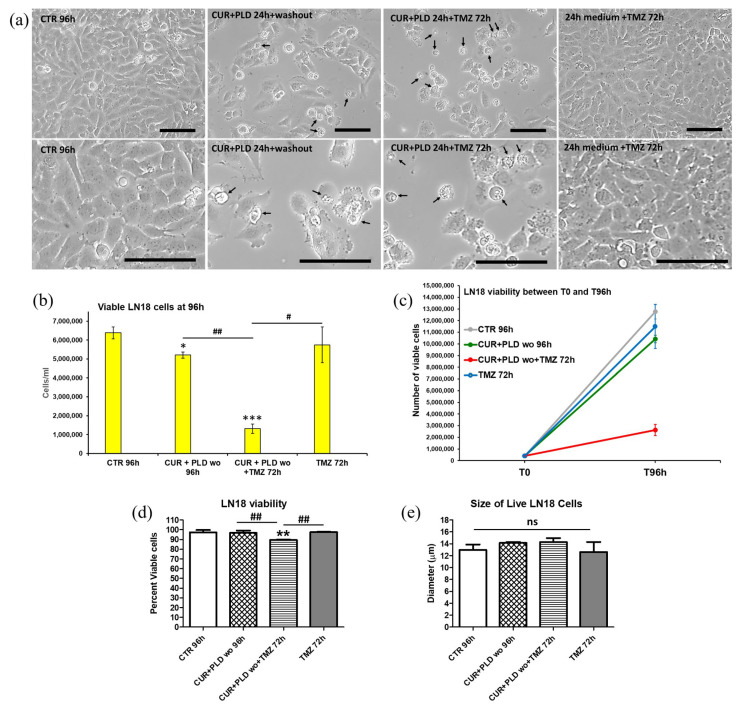
Effects of pretreatment with the synergic combination of CUR+PLD on TMZ-induced effects on LN18 cells. (**a**) Phase contrast microscopy images showing the morphological modification induced in untreated controls and in samples pretreated for 24 h with CUR+PLD with or without TMZ post-treatment. Arrows point to numerous cells displaying morphological features suggestive of apoptosis/autophagy. The scale bars represent 100 μm. (**b**–**e**) Analysis results from the automated CytoSmart cell counter: the number of live cells (**b**), cell growth between T0 and T 96 h (**c**), percent viable cells (**d**), and cell size (**e**). Results are the mean ± SD from three independent experiments (*n* = 3). Significance (one-way ANOVA + Tukey multiple comparison test): * *p* < 0.05, ** *p* < 0.01, *** *p* < 0.001 vs. untreated control (CTR); # *p* < 0.05, ## *p* < 0.01 vs. TMZ alone or pretreated with CUR+PLD; ns: not significant.

To verify the effects of the 24 h pretreatment with CUR+PLD on TMZ cytotoxicity, both cell lines were subjected to TMZ treatment and analyzed after an additional 72 h, following the experimental protocol schematized in Figure 2a. Phase contrast microscopy of the U87 cells showed that in both samples pretreated for 24 h with CUR+PLD (with or without TMZ post-treatment), the astrocyte-like morphology observed after 24 h of CUR+PLD was still preserved, and dying cells were almost absent (Figure 4a). The analyses by the automated CytoSmart cell counter found that the U87 viability was not affected (Figure 4d), while the number of live cells between T0 and 96 h was significantly reduced in all the treated samples compared with the untreated control (Figure 4b,c). Furthermore, in the sample pretreated with CUR+PLD followed by TMZ for 72 h, the number of live cells was significantly lower than that of the sample treated with TMZ alone. Interestingly, U87 cells treated with TMZ alone or combined with CUR+PLD were larger than the cells in the untreated and CUR+PLD-treated samples (Figure 4e). Conversely, in the LN18 cultures, which possess intrinsic resistance to TMZ because of the presence of MGMT gene transcripts, the TMZ treatment alone did not significantly affect the viability or the number of live cells compared with the untreated control. However, both parameters significantly decreased in the sample treated with CUR+PLD before TMZ (Figure 5b–d). In contrast to U87 cells, in LN18 cells treated with CUR+PLD alone or with TMZ, numerous cells displaying morphological features suggestive of apoptosis/autophagy induction were observed (arrows in Figure 5a), whereas the cell size was not significantly different (Figure 5e).

### 2.4. Pretreatment with CUR+PLD Induced a Cell Cycle Arrest between the S and G2/M Phases

**Figure 6:**
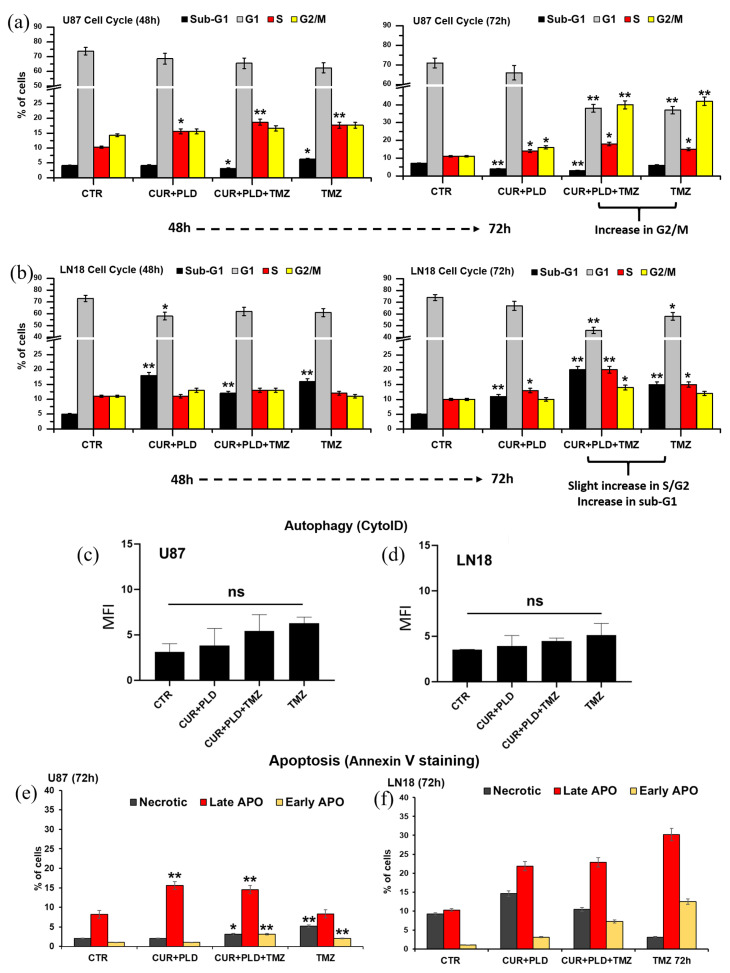
Effects of TMZ treatment with or without pretreatment with CUR+PLD on cell cycle, autophagic flux, and apoptosis. (**a**,**b**) The bar graphs illustrate the results of the cytofluorimetric analysis of the cell cycle in U87 (**a**) and LN18 (**b**) cells between 48 and 72 h of culture. (**c**,**d**) Cytofluorimetric analysis of autophagy performed using the Cyto-ID autophagy detection kit after 72 h of TMZ treatment in U87 (**c**) and LN18 (**d**) cells. (**e**,**f**) Cytofluorimetric evaluation of apoptosis using Annexin V (AV) staining in U87 (**e**) and LN18 (**f**) cells. PI−/AV+, PI+/AV+, and PI+/AV− counts indicate early apoptotic, late apoptotic, and necrotic cells, respectively. Significance vs control (two-tailed Student’s *t*-test): * *p* < 0.05, ** *p* < 0.01; ns: not significant.

The cytofluorimetric analysis of the cell cycle carried out on U87 cells recorded, in both TMZ-treated samples, an increase in the percentage of cells in the G2/M phase (about 40% of cells vs. 11% and 16% in the untreated control and CUR+PLD treatment alone, respectively) (Figure 6a and Supplementary Table S1). In LN18 cells, the analysis revealed a slight increase in the percentage of cells in the S/G2 phase in the sample pretreated with CUR+PLD plus TMZ compared to the other samples (20% vs. 10%, 13%, and 15% in the control, CUR+PLD, and TMZ alone, respectively) as well as an increase in the percentage of cells in the sub-G1 peak, which is typically associated with apoptotic/necrotic cells (20% vs. 5%, 11%, and 15% in the control, CUR+PLD, and TMZ alone, respectively) (Figure 6b and Supplementary Table S1). 

The flow cytometric monitoring of autophagy, using the CytoID kit (Figure 6c,d), and apoptosis, using annexin V/PI staining (Figure 6e,f, and Supplementary Table S2), showed that all the treatments did not significantly modify the autophagic flux in both cell lines; it very weakly (in U87 cells) or moderately (in LN18 cells) increased the percentage of cells in early/late apoptosis, consistent with the results obtained from the cell cycle analysis. Interestingly, in LN18 cells subjected to the CUR+PLD treatment, a low but significant increase in the percentage of necrotic cells (about 15% vs. 9% in the untreated control) was recorded, suggesting that CUR+PLD might cause secondary necrosis, triggered by the considerable level of caspase-dependent apoptosis, as measured by the level of PARP cleavage, after 24 h of the CUR+PLD pretreatment (Figure 3e).

### 2.5. Effects of the Synergic Combination of CUR+PLD on the Expression of MGMT and Proteins Related to Cell Proliferation, Apoptosis, and Autophagy

**Figure 7:**
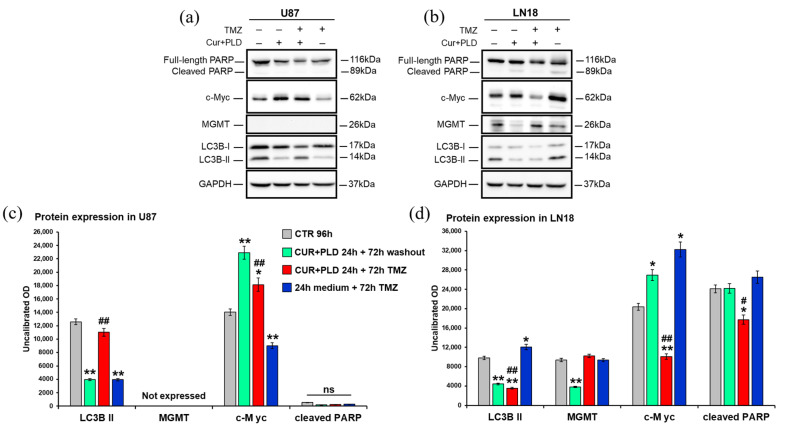
Effects on expression of MGMT and proteins related to cell proliferation, apoptosis, and autophagy. Western blot (**a**,**b**) and densitometric analysis (**c**,**d**) of the expression levels of MGMT, the proliferation marker c-Myc, the astroglial differentiation marker GFAP, the autophagosomal marker LC3B, and the marker of caspase-dependent apoptosis PARP, evaluated after 72 h of TMZ treatment with or without pretreatment with CUR+PLD in U87 (**a**,**c**) and LN18 (**b**,**d**) cells; values were normalized to GAPDH. Results are the mean ± SD from three independent experiments (*n* = 3). Significance (one-way ANOVA + Tukey multiple comparison test): * *p* < 0.05, ** *p* < 0.01 vs. untreated control (CTR), # *p* < 0.05, ## *p* < 0.01 vs. TMZ alone or pretreated with CUR+PLD; ns: not significant.

To obtain molecular evidence that supports the data obtained by the viability and flow cytometry assays, the expression levels of proteins related to cell proliferation (c-Myc), caspase-dependent apoptosis (cleaved PARP), and autophagy (LC3B) were assessed after 72 h of TMZ treatment, with or without CUR+PLD pretreatment, using WB (Figure 7). To verify if the pretreatment with CUR+PLD could affect the methylation level of the MGMT gene promoter and, therefore, the transcription of the MGMT gene, the MGMT expression level was also analyzed.

In the U87 cells (Figure 7a,c), the WB analysis showed that, in all the samples analyzed, the cleavage of PARP was not affected, which is in line with the cytofluorimetric data, and MGMT gene transcription was not activated, suggesting that the treatments did not modify the methylation levels of the MGMT promoter. Unexpectedly, at the time point analyzed, the levels of c-Myc were only significantly decreased in the sample treated with TMZ alone compared to the control, whereas CUR+PLD (with or without TMZ treatment) seemed to push cells toward proliferation. Furthermore, the expression of the autophagosomal marker LC3B-II decreased in the samples treated with CUR+PLD or TMZ alone compared with the control, while in the CUR+PLD plus TMZ sample, the LC3B-II expression was significantly higher than that of cells treated with TMZ alone. These data, apparently in contrast to those obtained by flow cytometry, could be due to the distinct features of the two methods: while CytoID is more sensitive to dynamic changes in autophagy and, therefore, gives a measure of autophagic flux, WB for LC3B-II provides a direct measure of autophagosome formation and, hence, more specifically quantifies autophagy induction.

In the LN18 cells (Figure 7b,d), the level of cleaved PARP was not modified except in the CUR+PLD plus TMZ samples, where it was down-regulated, suggesting that the moderate apoptosis recorded by flow cytometry could be due to a caspase-independent mechanism. Interestingly, in the LN18 cells, MGMT gene transcription was down-regulated in the sample treated with CUR+PLD alone, which seemed to affect the methylation levels of the MGMT promoter, thus decreasing the intrinsic resistance of these cells to TMZ. Furthermore, in LN18 cells, the pretreatment with CUR+PLD before TMZ down-regulated c-Myc expression, which, conversely, was upregulated in cells treated with TMZ alone. The expression of the autophagosomal marker LC3B-II, which was increased in TMZ-treated LN18 cells, was conversely decreased by the CUR+PLD pretreatment, suggesting that this synergic combination could inhibit the autophagy-mediated defense mechanism adopted by the cells to survive the TMZ-induced DNA damage [32].

### 2.6. Pretreatment with CUR+PLD Alters the Actin Network and Ultrastructural Morphology of U87 and LN18 Cells

Actin filaments are one of the main components of the cellular cytoskeleton, which regulates the migration and invasiveness of cancer cells, including glioblastoma cells [33,34]; TMZ has been found to moderately alter the actin cytoskeleton regulatory pathways [35]. Thus, we also preliminarily assessed if the pretreatment with CUR+PLD could amplify the effect of TMZ on the actin cytoskeleton.

**Figure 8:**
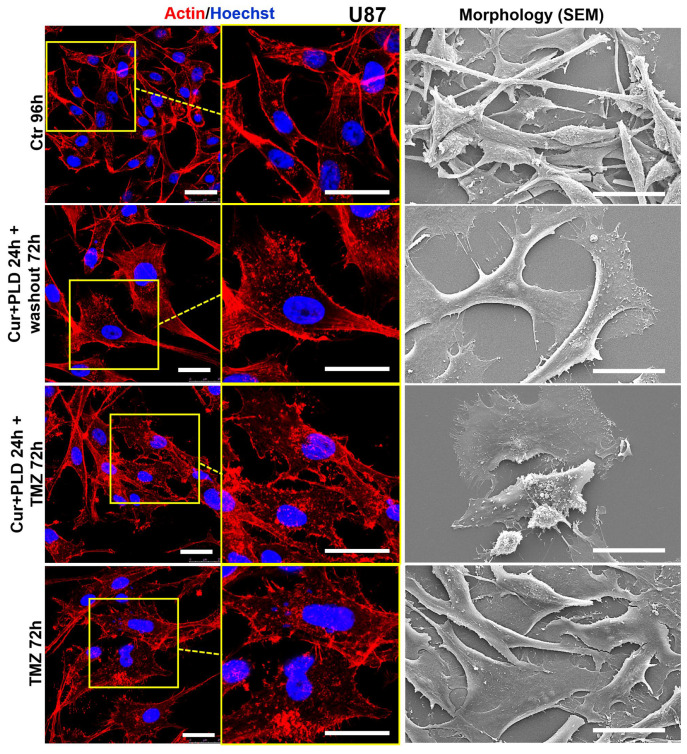
Effect of pretreatment with CUR+PLD on the actin network and ultrastructural morphology of U87 cells. Confocal microscopic analysis of the actin network (**left** panels) and SEM analysis of morphology (**right** panels), evaluated after 72 h of TMZ treatment with or without pretreatment with CUR+PLD. The scale bars represent 25 μm.

**Figure 9:**
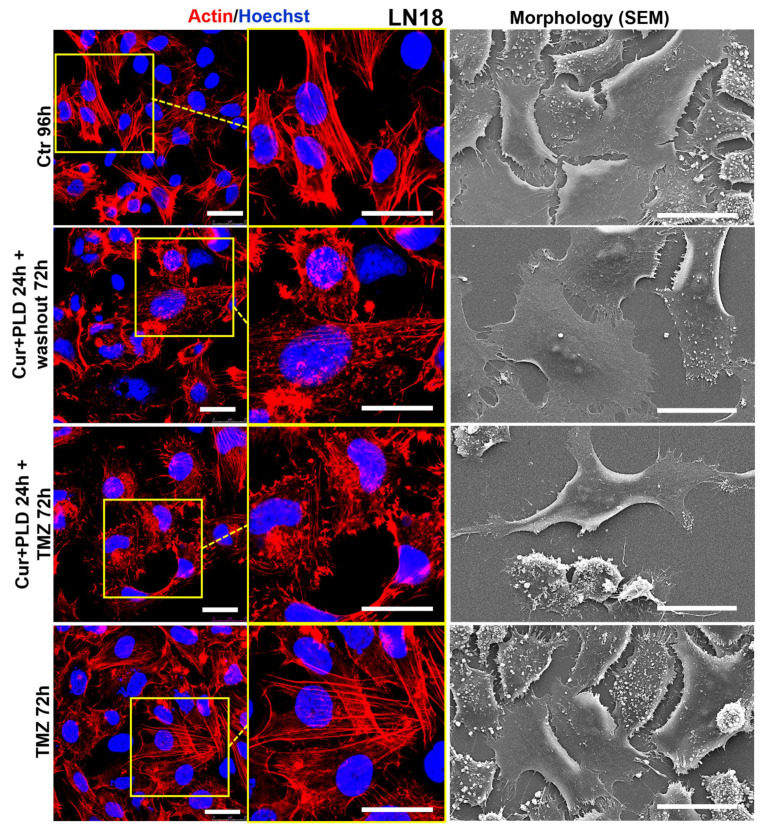
Effect of pretreatment with CUR+PLD on the actin network and ultrastructural morphology of LN18 cells. Confocal microscopic analysis of the actin network (**left** panels) and SEM analysis of morphology (**right** panels), evaluated after 72 h of TMZ treatment with or without pretreatment with CUR+PLD. The scale bars represent 25 μm.

Confocal microscopy (Figure 8 and Figure 9, left panels) showed that the control U87 and LN18 cells differed in organization and intracellular distribution of the actin network. Specifically, the actin cytoskeleton was less developed and the fibrillar form was mainly distributed at the cell edges and in the neurite-like structures in U87 cells (Figure 8, left panels), whereas it was highly polymerized, forming stress fibers inside almost all the cytoplasm in LN18 cells (Figure 9, left panels). These differences in the actin network are consistent with the different shapes of the two cell lines, as was shown by the SEM analysis, with the U87 cells being more elongated and the LN18 cells having a more polygonal and epithelial-like shape (Figure 8 and Figure 9, right panels). The TMZ treatment caused actin depolymerization and cell flattening in the more TMZ-sensitive U87 cells, whereas the actin network was less affected and the cell shape was quite similar to the untreated control in the intrinsically more resistant LN18 cells. Pretreatment with CUR+PLD before the TMZ treatment increased the TMZ-induced actin clumping in U87 cells, leading to partial detachment from the substrate (Figure 8). Interestingly, in LN18 cells, the CUR+PLD treatment alone induced significant actin depolymerization and cell flattening. This effect on the actin network was even more pronounced when the synergistic combination was used before the TMZ treatment, resulting in cells that appeared less adherent and partially elongated by SEM (Figure 9).

## 3. Discussion

Since ancient times herbal preparations have been used as the main source of therapeutic principles, and there are many remarkable examples in the history of medicine demonstrating how the discovery of natural products profoundly affected advances in biology and inspired drug discovery and therapy.

In recent decades, several naturally occurring dietary agents and a wide variety of plant-derived products have been recognized as promising candidates for developing chemopreventive or therapeutic drugs for cancer [4]. In particular, the anticancer activity of nature-derived polyphenols has been, and still is, the topic of numerous studies around the world [36]. Among polyphenols, CUR, RES, and more recently its glycoside PLD have been extensively investigated in preclinical in vitro and in vivo studies and have entered clinical trials [4,37,38]. The potential mechanisms underlying the anti-tumor efficacy of these nature-derived compounds involve their antioxidant and anti-inflammatory properties, as well as their ability to modulate the molecular pathways involved in cancer [4,36]. 

In this work, we showed that the pretreatment with a synergistic combination of CUR and PLD can increase the anti-tumor efficacy of TMZ in both GBL cell lines tested, as demonstrated by the results from the viability assays. However, the results obtained suggest that the mechanism of action underlying this efficacy might be different in the two cell lines, which possess different degrees of intrinsic sensitivity to TMZ treatment, as one is MGMT-positive with epithelial morphological characteristics (LN18) and the other is MGMT-negative with astroglial features (U87). Specifically, the first interesting result obtained was that the 24 h treatment (at T0 before the TMZ addition) with the selected synergistic combination of CUR+PLD was able to reduce cell proliferation in both tested GBL cell lines, as demonstrated by the decreased levels of c-Myc. However, the effect seemed to be mainly a consequence of the astroglial-like differentiation in the U87 cells, as suggested by the acquisition of a star-shaped morphology with extensive branching (Figure 3a) and the increase in GFAP expression (Figure 3b) [39,40], whereas the effect could possibly derive from the induction of apoptosis/autophagy in the intrinsically more resistant LN18 cells. These different effects exerted by the combination of the two compounds are in line with the widely described effects of CUR, PLD, as well as RES on the molecular pathways involved in numerous biological processes, including cell viability, survival, proliferation, differentiation, and apoptosis, such as the Notch and the Wnt/β-catenin pathways [4,41,42]. These pathways are known to be differently dysregulated in tumors depending on the cancer cells, and molecules targeting them can revert cancer cell malignancy by affecting distinct processes.

Even more interesting results were those that demonstrated that the pretreatment with CUR+PLD significantly improved the effect of the TMZ treatment on the number of live cells in both glioblastoma lines, and this effect was more evident in the intrinsically resistant LN18 cells, in which, TMZ alone was ineffective. The data from the cytofluorimetric evaluation of autophagic flux, apoptosis, and cell cycle showed that after 72 h of treatment with TMZ, in the presence or the absence of a CUR+PLD pretreatment, the autophagic flux was not modified in both cell lines. Apoptosis induction was recorded for both experimental conditions only in LN18 cells, but a cell cycle arrest between the S and G2/M phases was recorded in both cell lines. It has been reported that TMZ can cause a shift of glioblastoma cells into G2/M, a prosurvival strategy that the cells adopt to have time to repair the TMZ-induced DNA damage, and that the co-treatment with RES forces cells through mitosis, leading to mitotic catastrophe and senescence, thus increasing the chronic effects of TMZ [43]. Our WB results suggest that this is probably what occurred in U87 cells subjected to CUR+PLD plus TMZ, in which, concomitant with the recorded arrest in G2/M phase, the levels of the proliferation marker c-Myc and the autophagosomal marker LC3B-II were unexpectedly higher than in cells treated with TMZ alone. Instead, in CUR+PLD-pretreated U87 cells, the increased c-Myc levels observed after the 72 h washout period could be due to a gradual reversion of the astrocyte-like differentiation towards the undifferentiated and proliferative baseline condition once the pharmacological pressure was removed, as suggested by the decrease in the expression of the astroglial differentiation marker GFAP that was recorded at the same time point in our preliminary experiments. A different mechanism seems to be involved in the LN18 cells pretreated with CUR+PLD plus TMZ since the expression of the proliferation marker was lower than that in cells treated with TMZ alone, and autophagy appeared to be inhibited by the pretreatment. This result suggests that, in LN18 cells, PLD, similar to what was reported for its “twin” compound RES [44], suppresses the cytoprotective autophagy that occurs as a consequence of TMZ-induced DNA damage, thus improving the cytotoxicity of the chemotherapeutic drug.

Finally, the preliminary data on the actin network suggest that the synergic combination could also improve the described effect of TMZ (and other cytotoxic agents that induce DNA damage) on glioblastoma cell motility and invasiveness [35] and this effect is more marked in the intrinsically more resistant LN18 cells. Indeed, changes in actin cytoskeleton dynamics are crucial in regulating cell shape and motility and are involved in cancer cell migration and tumor progression. It has been reported that CUR might inhibit cancer cell motility by altering actin polymerization, possibly through direct interaction with this cytoskeleton element [45,46]. Furthermore, PLD can repress glioblastoma cell migration and invasiveness by inhibiting the EGFR/AKT/ERK1/2/STAT3/SOX2/Snail signaling pathway [24]. Our preliminary data on the actin network support these CUR and PLD properties and stimulate further studies focused on the effect of the synergic combination CUR+PLD on GLB cell migration and invasiveness.

The main limitation of our study lies in the fact that, being an in vitro study, it did not consider the concerns related to the bioavailability of the two natural compounds in vivo. Nevertheless, these difficulties are limited by (i) the higher solubility and bioavailability demonstrated in the literature for polydatin compared to resveratrol [14,15]; (ii) the increased water solubility of the formulation of curcumin that was used, which was specifically designed to enhance the water dispersibility of its bioactive compound; and (iii) the availability of an orodispersible nutraceutical formulation of CUR+PLD that can be administered to patients and can allow high blood levels of the compounds to be reached in a very short time, thus increasing their bioavailability [31]. Anyway, our results are very stimulating for future studies dedicated to exploring some aspects of the described effects in more depth, by investigating the involvement of relevant tumor-related pathways that are known to be affected by curcumin and polydatin (or its derivative resveratrol) such as the Notch and Wnt/β-catenin pathways [4,41,42].

## 4. Materials and Methods

### 4.1. Reagents

Temozolomide (TMZ, 3,4-dihydro-3-methyl-4-oxoimidazo [5,1-d]-astetrazine- 8-carboxamide) (Sigma-Aldrich, St. Louis, MO, USA; purity ≥ 98%; Cas: 85622-93-1) was dissolved in dimethyl sulfoxide (DMSO, Sigma-Aldrich) and stored as a 20 mM stock solution. A curcumin extract (CUR) and polydatin (PLD) were kindly donated by Sherman Tree Nutraceuticals s.r.l. (Rome, Italy). The CUR contained more than 85% curcumin by weight and was specifically designed to enhance the water dispersibility of its bioactive compound; the PLD has a purity of ≥98%. CUR and PLD were dissolved in DMSO and stored as a 10 mg/mL stock solution.

### 4.2. Cell Cultures

The human glioblastoma cell lines U87 and LN18 were originally obtained from the American Tissue Culture Collection (ATCC, Manassas, VA, USA). An aliquot of each cell line was revived for each experiment and then maintained in culture for up to 2 months before use. All the experiments were performed on cells between the 7th and 9th passages. Both the U87 and LN18 cells were grown as monolayers in Dulbecco’s Modified Eagle Medium (DMEM) plus HAM’s F12 (in 1:1 ratio), supplemented with 10% heat-inactivated fetal bovine serum (FBS), L-glutamine (2 mM), penicillin (100 IU/mL), and streptomycin (100 µg/mL), and maintained at 37 °C in a 5% CO_2_, humidified atmosphere. For passaging, cells were detached with a 0.05% trypsin and 0.002% EDTA solution. All media and supplements for the cell cultures were acquired from Gibco (Thermo Fisher Scientific, Inc., Waltham, MA, USA).

### 4.3. Cell Treatments and Cell Viability Analyses

In the preliminary dose–response experiments, U87 and LN18 cells were seeded in 96-well plates (5 × 10^3^ cells/well), and treated after 24 h of adhesion with TMZ (from 50 to 400 µM), or with CUR (from 0.18 to 10 µg/mL) and PLD (from 17.5 to 300 µg/mL) alone or in combination, for 24, 48, and 72 h. At the end of the incubation period, the cells were incubated with a 1 mg/mL 3-(4,5-dimethylthiazol-2-yl)-2,5-diphenyltetrazolium bromide (MTT) solution (Sigma-Aldrich) for 3 h at 37 °C. The supernatants were removed from the wells and formazan was solubilized with 150 μL of DMSO. The absorbance was read at 570 nm using a Multiskan Labsystem microplate reader (Thermo Fischer Scientific). 

To determine the synergistic effects of CUR and PLD, the CalcuSyn software (Biosoft v2.1) was used to calculate the combination index (CI). The concentrations of 10 µg/mL for CUR and 100 µg/mL for PLD (ratio of 1:10) were established as those that produced synergism in both cell lines (CI < 0.9).

Treatments were performed following the scheme reported in Figure 2a. Briefly, both cell lines were pre-incubated with CUR+PLD for 24 h before the TMZ treatment, and analyzed after 24 h (T0 before TMZ treatment) and after an additional 72 h in terms of cell morphology and viability, and the expression levels of MGMT and proteins related to cell proliferation, differentiation, apoptosis, and autophagy. The effects on the actin network were also analyzed by confocal microscopy. The trypan blue dye exclusion method, followed by analysis using an automated CytoSmart cell counter (Corning, Glendale, AZ, USA), was used in all the experiments performed to determine cell viability after the CUR+PLD plus TMZ combined treatments. CytoSmart was also used to automatically obtain a measure of cell size for the treated and untreated samples, in parallel to the cell count and viability measurements.

### 4.4. Evaluation of Cell Morphology

The morphological features of the treated and untreated cells were analyzed by phase contrast microscopy using the Motic AE31 Trinocular inverted microscope (Motic Asia, Hong Kong, China). Scanning electron microscopy (SEM) was also used to analyze the ultrastructural features in the control and treated cultures. Specifically, for the SEM analyses, U87 and LN18 cells were cultured on 12 mm glass coverslips and treated as schematized in Figure 2a. After 72 h of TMZ treatment, the cells were fixed in 2.5% glutaraldehyde in 0.2 M Na-cacodylate buffer (pH 7.4) for 2 h at room temperature. Following three washes with the same buffer, the cells were fixed with 1% (*w*/*w*) OsO_4_ for 1 h, dehydrated through an ethanol gradient, and treated to dry using hexamethyldisilazane (HDMS, Sigma-Aldrich). The samples were coated with gold using a sputter coater and analyzed using the FEI Quanta Inspect FeG scanning electron microscope (FEI Company, Eindhoven, The Netherlands).

### 4.5. Western Blot (WB) Analysis

The cells were lysed in RIPA lysis buffer containing 50 mM Tris/HCl (pH 8.0), 150 mM NaCl, 1% NP40 nonidet, 0.5% sodium deoxycholate, 0.1% SDS, 1 mM PMSF, 2 mM Na_3_VO_4_, 20 mM NaF, and 1% protease inhibitor cocktail (Sigma-Aldrich). Lysates were clarified by centrifugation, and the protein content was determined using the Bradford reagent (Bio-Rad, Segrate, Italy). A total of 15–20 µg of each cell extract was separated on a 10–15% gel by SDS-PAGE, transferred to a nitrocellulose membrane (Hybond; Amersham GE Healthcare, Chicago, IL, USA), blocked with 5% non-fat milk (Bio-Rad) in Tris-buffered saline with Tween (TBS-T; 20 mM Tris, 150 mM NaCl (pH 7.6), and 0.05% Tween-20) and probed with primary antibodies against MGMT, c-Myc, LC3B, PARP, and GFAP, as detailed in Supplementary Table S3. The primary antibodies were detected using peroxidase-conjugated secondary antibodies (Bio-Rad, Richmond, CA, USA). The ChemiDoc XRS+ imager (Bio-Rad) was used for membrane exposure and image acquisition. The densities of the bands were quantified using the ImageJ processing software (version 1.52p). The values, normalized to GAPDH, are reported as uncalibrated Optical Density (OD) values or the fold change vs. untreated control. The results were from at least three independent experiments and are presented as the mean ± SD.

### 4.6. Confocal Laser Scanning Microscopy (CLSM)

For confocal microscopy, the cells were fixed with 2% paraformaldehyde (Sigma-Aldrich) for 20 min, permeabilized with 0.2% Triton X-100 (Sigma-Aldrich) for 4 min, and stained with Alexa Fluor 546-conjugated phalloidin (Thermo Fisher Scientific; working dilution 1:40) for 30 min. Nuclei were counterstained with Hoechst 33285 (Fluka Biochemika, Buchs, Switzerland, working dilution 1:3000) for 5 min. The analyses were carried out using the Stellaris 5 confocal microscope from LEICA Microsystems (Wetzlar, Germany).

### 4.7. Cytofluorimetric Analyses of Cell Cycle, Apoptosis, and Autophagy

The cell cycle was analyzed in U87 and LN18 cells after 48 and 72 h of TMZ treatment in the absence or the presence of a CUR+PLD pretreatment. At these time points, the cells were collected, washed with cold PBS, and fixed in 70% ethanol for 1 h at 4 °C. After washing with cold PBS, the cells were resuspended in 40 µg/mL propidium iodide (PI) and 100 µg/mL RNAse in PBS at 37 °C for 1 h, and the percentages of cells in the different phases were determined by flow cytometry.

The assessment of apoptosis was performed using a fluorescein isothiocyanate (FITC)-conjugated annexin V (AV) and PI detection kit according to the manufacturer’s protocol (BioVision Incorporated, Milpitas, CA, USA). Untreated controls and treated cells were collected after 72 h, washed with PBS, and resuspended at a concentration of 2.5 × 10^5^ cells/mL. Subsequently, 5 μL of an annexin V-FITC solution and an equal volume of a PI solution were added to 500 μL of the cell suspension, incubated for 5 min at room temperature in the dark, and analyzed by flow cytometry.

The monitoring of autophagic flux was performed on live cells using the Cyto-ID Autophagy Detection Kit (Enzo Life Sciences, Farmingdale, NY, USA) according to the manufacturer’s instructions. Briefly, untreated controls and treated cells were collected after 72 h, stained with the Cyto-ID staining solution for 30 min at 37 °C, and the fluorescence intensities were determined by flow cytometry. For all the analyses, the samples were analyzed using a flow cytometry instrument (Gallios Instrument, Beckman Coulter, Brea, CA, USA), and at least 10,000 events were run per sample. The data were analyzed using Kaluza software v. 2.2 (Beckman Coulter, Brea, CA, USA). The gating strategies used for the cell cycle, autophagy, and apoptosis evaluations are shown in Supplementary Figures S2–S4.

### 4.8. Statistical Analysis

Statistical analysis was performed using the two-tailed Student’s *t*-test or one-way analysis of variance (ANOVA), followed by the Tukey post hoc test to analyze the differences between groups. A *p*-value < 0.05 was assumed to be statistically significant. All results were from at least three independent experiments and are presented as the mean ± SD.

## 5. Conclusions

The results obtained in this work provide convincing evidence that CUR and PLD, by acting in synergy with each other, combine their abilities to affect several processes crucial for the viability, survival, differentiation, and motility of tumor cells, and might strongly improve the efficacy of alkylating anti-tumor agents such as TMZ in drug-resistant cells, and are stimulating for further studies to deepen our knowledge of some aspects of the described effects. The data also provide experimental evidence reinforcing the beneficial effects of the synergic combination of CUR+PLD that have been recorded in glioblastoma patients [31], supporting the use of this orodispersible nutraceutical formulation in integrated anticancer therapies. The demonstrated efficacy in different GBL cell lines that exhibit different phenotypical features and different degrees of TMZ sensitivity suggest that the use of CUR+PLD as an integrated therapy might be beneficial in poorly differentiated and multifaceted tumors such as glioblastoma multiforme. Furthermore, owing to the demonstrated ability of CUR+PLD in improving TMZ cytotoxicity in intrinsically resistant MGMT+ cells, the synergic combination of these two natural molecules could also be beneficial in other tumors in which the treatment with alkylating agents are often ineffective because of the presence of high levels of MGMT, such as pancreatic tumors. This assumption is supported by our preliminary clinical data suggesting that the CUR+PLD combination could also lead to positive results in pancreatic cancer, which is the subject of our ongoing clinical investigations.

## Data Availability

All the data produced in this study are reported in this article. The primary data files are available from the corresponding author upon reasonable request.

## Supplementary Materials

The following supporting information can be downloaded at: https://www.mdpi.com/article/10.3390/ijms251910572/s1.
