# Supplementary material for: The Synergistic Combination of Curcumin and Polydatin Improves Temozolomide Efficacy on Glioblastoma Cells
Source: Int J Mol Sci. 2024 Sep 30;25(19):10572. doi: 10.3390/ijms251910572 (PMC11477178; doi:10.3390/ijms251910572)
Supplement: Supplementary file 1 [file ijms-25-10572-s001.zip › ijms-3165330-supplementary.pdf]

The synergistic combination of curcumin and polydatin improves temozolomide efficacy on glioblastoma cells

Annalucia Serafino, Ewa K. Krasnowska, Sabrina Romanò, Alex De Gregorio, Marisa Colone, Maria Luisa Dupuis, Massimo Bonucci, Giampietro Ravagnan, Annarita Stringaro & Maria Pia Fuggetta

SUPPLEMENTARY FIGURE AND TABLES

Data from CALCUSYN software

(a)

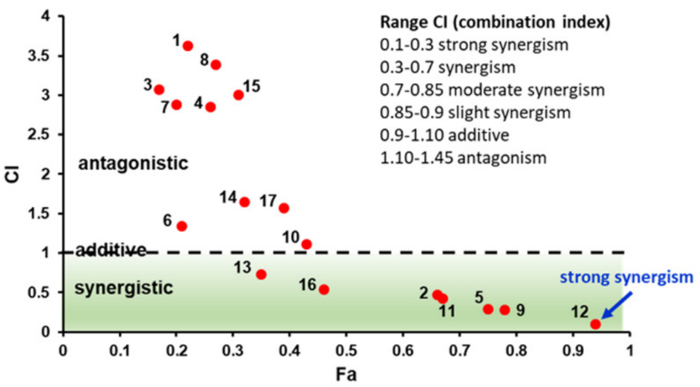

CI values for treatments on U87 cells

Comb #	PLD (µg/ml)	CUR (µg/ml)	CR	Fa	CI
1	8.75	5	1.75:1	0.216	3.626
2	17.5	10	1.75:1	0.661	0.467
3	8.75	2.5	3.5:1	0.171	3.074
4	17.5	5	3.5:1	0.262	2.85
5	35	10	3.5:1	0.747	0.292
6	8.75	1.25	7 :1	0.208	1.344
7	17.5	2.5	7 :1	0.2	2.882
8	35	5	7 :1	0.267	3.388
9	70	10	7 :1	0.777	0.282
10	35	3.5	10:1	0.435	1.11
11	70	7	10:1	0.669	0.426
12	100	10	10:1	0.942	0.101
13	17.5	1.25	14:1	0.335	0.731
14	35	2.5	14:1	0.317	1.643
15	70	5	14:1	0.331	3.002
16	35	1.25	28:1	0.458	0.537
17	70	2.5	28:1	0.393	1.570

CR: COMBINATION RATIO
CI: COMBINATION INDEX
Fa: FRACTION AFFECTED (percent of inhibition/100)

(b)

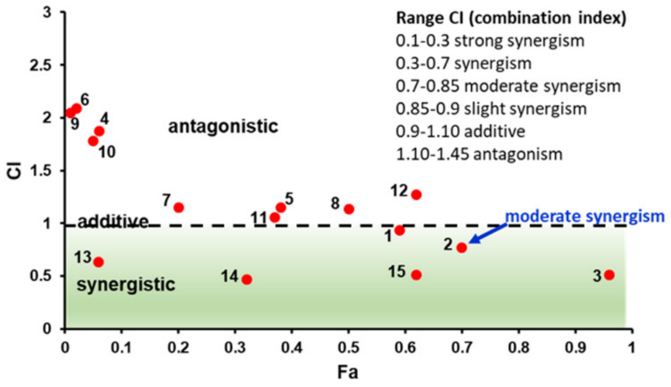

CI values for treatments on LN18 cells

Comb #	PLD (µg/ml)	CUR (µg/ml)	CR	Fa	CI
1	75	7.5	10:1	0.589	0.935
2	100	10	10:1	0.697	0.772
3	150	15	10:1	0.965	0.514
4	37.5	1.5	25:1	0.06	1.873
5	75	3	25:1	0.38	1.148
6	37.5	0.75	50:1	0.02	2.092
7	75	1.5	50:1	0.2	1.153
8	150	3	50:1	0.5	1.134
9	37.5	0.37 5	100:1	0.01	2.048
10	75	0.75	100:1	0.05	1.780
11	150	1.5	100:1	0.37	1.059
12	300	3	100:1	0.62	1.269
13	37.5	0.18	200:1	0.06	0.630
14	75	0.375	200:1	0.32	0.471
15	150	0.75	200:1	0.62	0.510

CR: COMBINATION RATIO
CI: COMBINATION INDEX
Fa: FRACTION AFFECTED (percent of inhibition/100)

Figure S1. Representative data from CalcuSyn software used to define the synergic concentrations of CUR and PLD in U87 (a) and LN18 (b) glioblastoma cells. Left panels report the CI-Fa plot in which values for synergistic, additive and antagonistic concentrations of CUR and PLD are illustrated; arrows point to the synergic combinations selected for the experiments in the two cell lines. The right tables report the most representative values of CUR and PLD concentrations and combination ratios (CR), faction affected (Fa) and combination index (CI) from which the left plots are obtained; the CI of the selected synergic combinations are marked in red.

The synergistic combination of curcumin and polydatin improves temozolomide efficacy on glioblastoma cells

Annalucia Serafino, Ewa K. Krasnowska, Sabrina Romanò, Alex De Gregorio, Marisa Colone, Maria Luisa Dupuis, Massimo Bonucci, Giampietro Ravagnan, Annarita Stringaro & Maria Pia Fuggetta

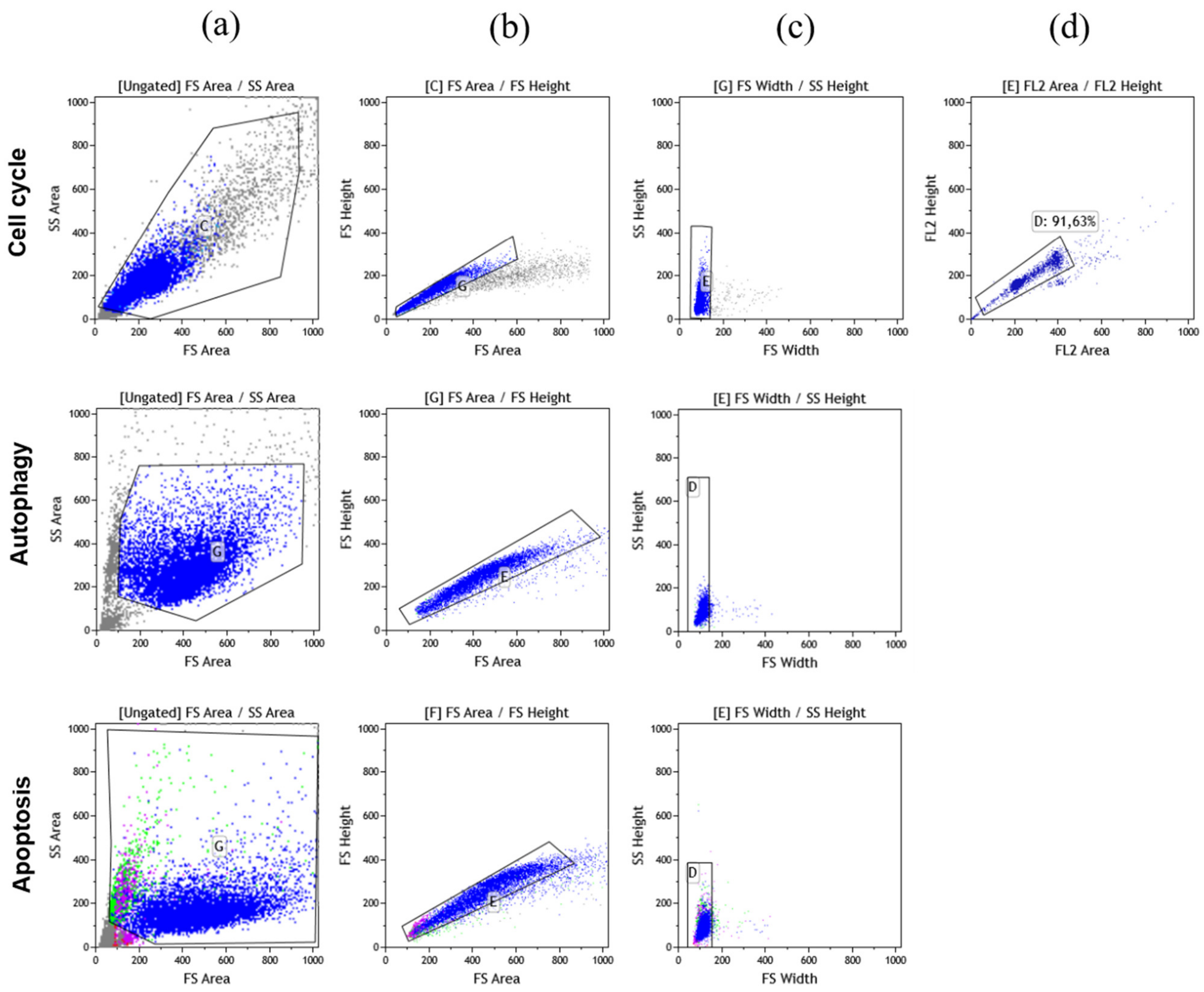

Figure S2. Gating strategy of the cell population for cell cycle, autophagy, and apoptosis study in cytofluorimetry. Cells were first gated on forward scattering area (FSC-A) versus side scattering area (SSC-A) signals to exclude debris (Panel a). FSC-A versus FSC height (Panel b) and FSC width versus SSC height (panel c) gating strategies were used to exclude doublet events and cell clumps. In paned (d), FL-2 area versus FL-2 height gate dot plot was used to define the total cell population in the different phases of the cell cycle.

The synergistic combination of curcumin and polydatin improves temozolomide efficacy on glioblastoma cells

Annalucia Serafino, Ewa K. Krasnowska, Sabrina Romanò, Alex De Gregorio, Marisa Colone, Maria Luisa Dupuis, Massimo Bonucci, Giampietro Ravagnan, Annarita Stringaro & Maria Pia Fuggetta

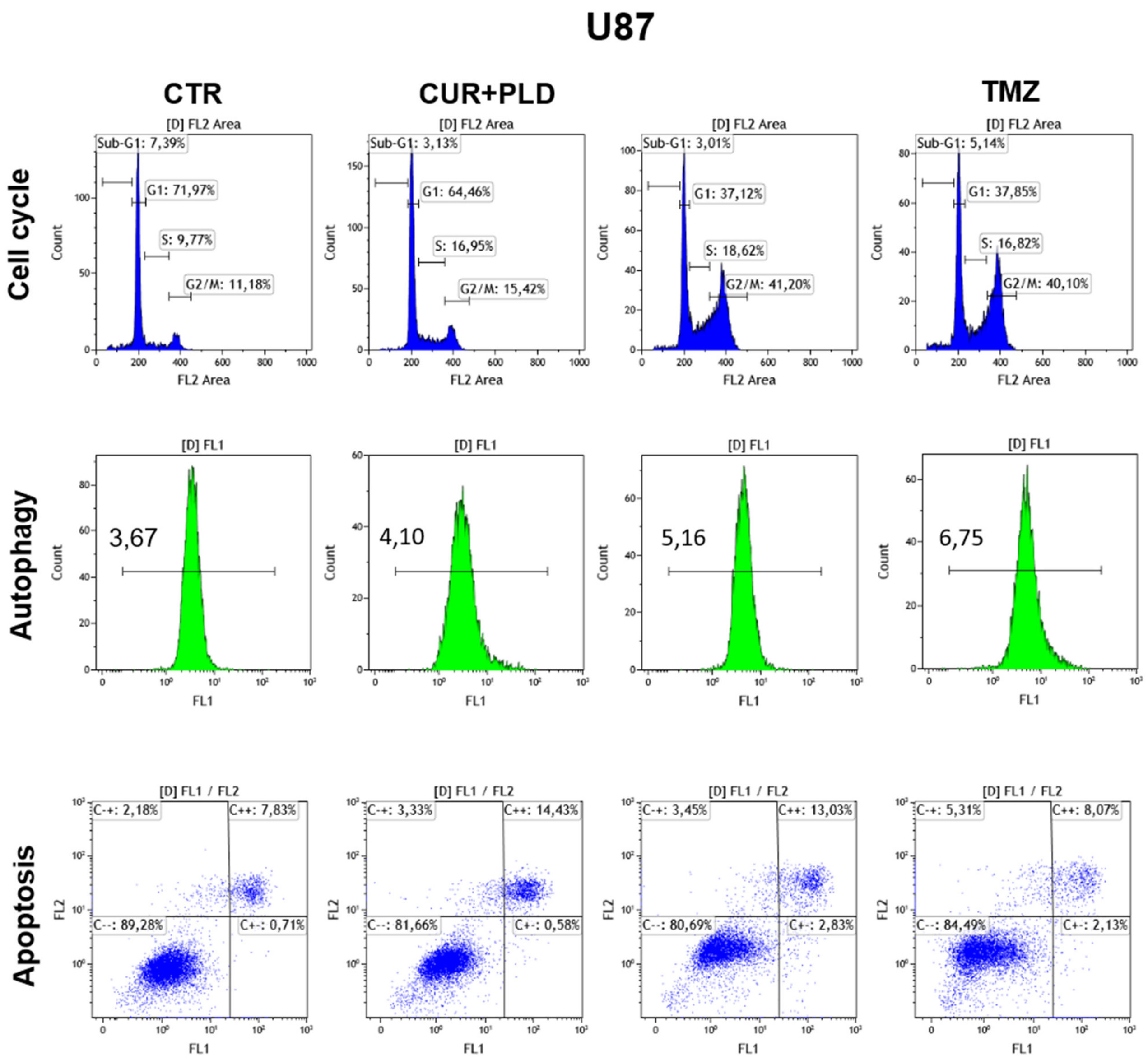

Figure S3. Effects of the TMZ treatment with or without pretreatment with CUR+PLD on cell cycle, autophagy, and apoptosis. Representative histograms and dot plots of results obtained in U87 cell line treated or untreated with TMZ and CUR+PLD for 72h.

The synergistic combination of curcumin and polydatin improves temozolomide efficacy on glioblastoma cells

Annalucia Serafino, Ewa K. Krasnowska, Sabrina Romanò, Alex De Gregorio, Marisa Colone, Maria Luisa Dupuis, Massimo Bonucci, Giampietro Ravagnan, Annarita Stringaro & Maria Pia Fuggetta

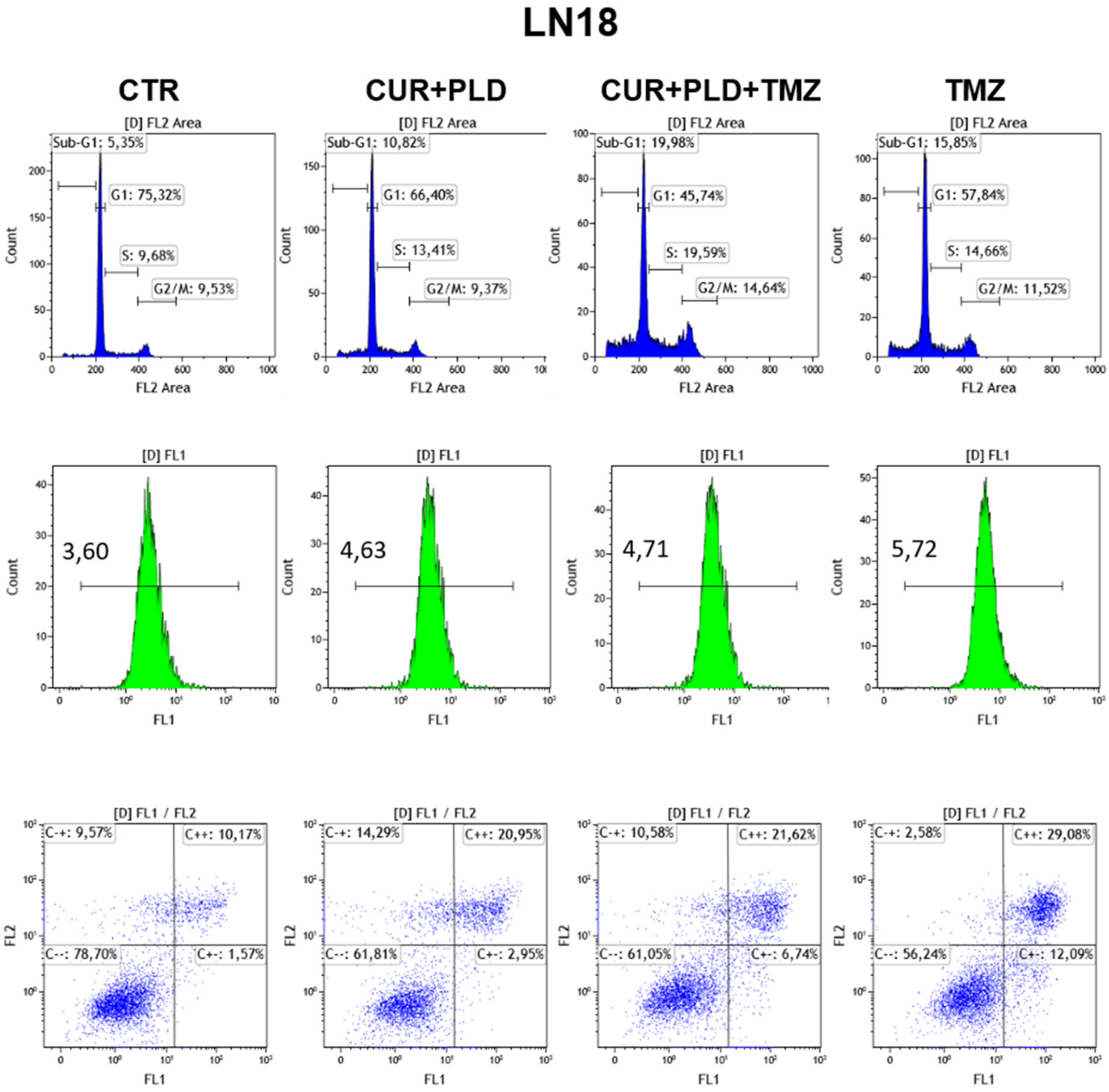

Figure S4. Effects of the TMZ treatment with or without pretreatment with CUR+PLD on cell cycle, autophagy, and apoptosis. Representative histograms and dot plots of results obtained in LN18 cell line treated or untreated with TMZ and CUR+PLD for 72h.

The synergistic combination of curcumin and polydatin improves temozolomide efficacy on glioblastoma cells

Annalucia Serafino, Ewa K. Krasnowska, Sabrina Romanò, Alex De Gregorio, Marisa Colone, Maria Luisa Dupuis, Massimo Bonucci, Giampietro Ravagnan, Annarita Stringaro & Maria Pia Fuggetta

Table S1. Data from cytofluorometric analysis of cell cycle in U87 and LN18 glioblastoma cells. Values increased by the treatments are marked in red

U87 cells	Sub-G1	G1	S	G2/M
CTR	7	71	11	11
CUR+PLD 24h + 72h fresh-medium	4	66	14	16
CUR+PLD 24h + TMZ 72h	3	38	**18**	**40**
TMZ 72h	6	37	15	**42**
**LN18 cells**	**Sub-G1**	**G1**	**S**	**G2/M**
CTR	5	74	10	10
CUR+PLD 24h + 72h fresh-medium	11	67	13	10
CUR+PLD 24h + TMZ 72h	**20**	46	**20**	**14**
TMZ 72h	**15**	58	**15**	12

Table S2. Data from cytofluorometric analysis of apoptosis/necrosis by Annexin V and propidium iodide (PI) staining in U87 and LN18 glioblastoma cells. Values increased by the treatments are marked in red

U87 cells	% of Necrotic cells ($Annex^-/PI^+$)	% of Late apoptotic cells ($Annex^+/PI^+$)	% of Early apoptotic cells ($Annex^+/PI^-$)
CTR	2	8	1
CUR+PLD 24h + 72h fresh-medium	2	**15**	1
CUR+PLD 24h + TMZ 72h	3	**14**	3
TMZ 72h	5	8	2
**LN18 cells**	**% of Necrotic cells (PI+/Annex-)**	**% of Late apoptotic cells (Annex+/PI+)**	**% of Early apoptotic cells (Annex+/PI-)**
CTR	9	10	1
CUR+PLD 24h + 72h fresh-medium	**14**	**21**	3
CUR+PLD 24h + TMZ 72h	**10**	**22**	7
TMZ 72h	3	**29**	**12**

Table S3. List of antibodies/reagents used for Western blot analyses.

Antigen	Host	Cat. #	Working dilution	Supplier
**MGMT**	Mouse (monoclonal)	35-7000	1:500	Thermofisher Scientific
**c-Myc**	Rabbit (monoclonal)	32072	1:1000	Abcam
**LC3B**	Rabbit (monoclonal)	3868	1:1000	Cell Signalling Technology
**PARP (total and cleaved)**	Rabbit (polyclonal)	9542	1:1000	Cell Signalling Technology
**GFAP**	Mouse	25778	1:1000	BD Biosciences
**GAPDH**	Rabbit (polyclonal)	25778	1:15000	Santa Cruz
